# Optical and Electrical Properties of Boron-Based Low-Dimensional Nanomaterials

**DOI:** 10.3390/nano16120723

**Published:** 2026-06-11

**Authors:** Jumpei Kawaguchi, Tetsuya Kambe

**Affiliations:** 1 Division of Applied Chemistry, Graduate School of Engineering, The University of Osaka, 2-1 Yamadaoka, Suita 565-0871, Japan; junpei_kawaguchi@chem.eng.osaka-u.ac.jp; 2 Department of Chemistry, Institute of Pure and Applied Sciences, University of Tsukuba, 1-1-1 Tennodai, Tsukuba 305-8571, Japan; 3 Tsukuba Institute for Advanced Research (TIAR), University of Tsukuba, 1-1-1 Tennodai, Tsukuba 305-8571, Japan

**Keywords:** boron, nanomaterials, properties

## Abstract

Low-dimensional (0D/1D/2D) nanomaterials exhibit unique physical and chemical properties different from general bulk materials due to enhanced surface and interface contributions and quantum confinement effects, which strongly modulate electronic structures. Boron, with atomic number 5, can form multicenter bonds and enables the construction of structurally diverse nanomaterials across different dimensionalities. In this review, boron-based low-dimensional materials are systematically organized from 0D clusters to 1D nanostructures and 2D sheets, and their optical and electrical properties are discussed in relation to structural factors such as dimensionality. This review provides an integrated perspective on how dimensional expansion and structural design govern the optical and electrical properties of boron-based nanomaterials.

## 1. Introduction

Low-dimensional nanomaterials are known to exhibit attractive physical and chemical properties fundamentally different from corresponding bulk materials [1,2,3]. Reduction in dimensionality enhances the contribution of surfaces and interfaces, leading to significant changes in material properties. Furthermore, quantum confinement effects acting on delocalized electrons can modulate electronic structures and excited-state behaviors. From the perspective of optical functionality, reduced dimensionality often influences light absorption, emission behavior, exciton formation, and carrier relaxation processes [4]. From the perspective of electrical functionality, charge transport properties, band alignment, contact resistance, and defect-mediated conduction are strongly dependent on structural factors associated with reduced dimensionality [5,6]. Although optical and electrical properties are sometimes interrelated, it is important to systematically understand them based on common structural factors.

**Figure 1:**
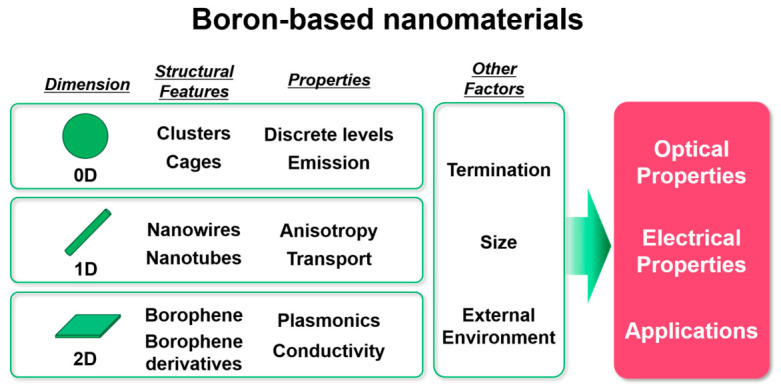
Conceptual overview of boron-based low-dimensional materials.

Boron, as a building unit of low-dimensional materials, is an element with three valence electrons which can form multicenter bonds. The property provides a high degree of structural flexibility [7,8]. This feature enables the formation of a variety of low-dimensional structures, including 0D clusters, 1D nanowires and nanotubes, and 2D borophene and the derivatives, which have been investigated theoretically or experimentally [9,10,11]. However, challenges such as chemical stability, mass synthesis, and interface control remain. In this review, boron-based low-dimensional materials are organized according to dimensionality. Their optical and electrical properties are discussed in an integrated manners in relation to structural factors such as termination, size, and external environments (Figure 1).

## 2. Zero-Dimensional Materials

Among boron-based low-dimensional materials, boron clusters represent zero-dimensional systems with finite molecular frameworks and discrete electronic states. In this section, borane clusters and all-boron fullerenes are discussed, with emphasis on how cage topology, substituent effects, symmetry, and chirality govern their optical, redox, and transport-related properties.

### 2.1. Borane Clusters

Closo-borane-type clusters (B_n_H_n_^2−^) with polyhedral structures are representative zero-dimensional boron materials that have been extensively studied both theoretically and experimentally since their first synthesis reports in 1959 [12,13,14,15,16,17,18,19]. These clusters form polyhedral frameworks through multicenter bonding arising from electron deficiency and exhibit high stability based on σ-aromaticity within the cluster. Therefore, these materials are easy to handle experimentally, and numerous experimental studies have been conducted to date. In addition, closo-borane-type clusters can form a wide variety of derivatives through substitution of terminal hydrogen atoms or cage atoms. For example, substitution of [BH] vertices with isoelectronic [CH^+^] units yields carboranes, which can further develop into metallacarboranes through metal coordination. Such chemical modifications enable systematic tuning of electronic structures and properties.

**Figure 2:**
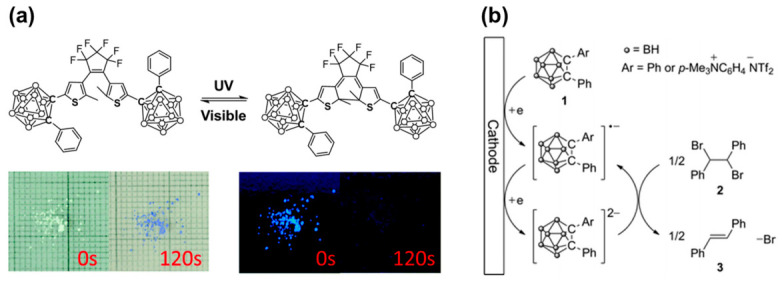
(**a**) Photochromic behavior of dithienylethene derivatives and photoswitch under white light (right) and UV light (left). The length of time taken in seconds after UV irradiation (0.96 mW·cm^−2^, 302 nm) is indicated. Adapted from Ref. [25] under the terms of the Creative Commons Attribution 3.0 Unported Licence. (**b**) proposed catalytic cycle for the indirect electrochemical reduction of a substrate using carboranes. Adapted with permission from Ref. [29]. Copyright 2011, Royal Society of Chemistry.

From the viewpoint of optical properties, luminescence in carborane derivatives has been reported since 2004 [20,21]. This emission behavior is associated with the electron-deficient boron-rich cage of carborane, which can function as an electron-accepting unit. When electron-donating aromatic groups are introduced, donor–acceptor structures are formed, and emission originating from intramolecular charge transfer (ICT) excited states is observed [22,23]. In phenyl-substituted derivatives, dual emission around 505 nm has been reported [24], and dithienylethene derivatives exhibit photochromic behavior and applications in organic light-emitting diodes (Figure 2a) [25,26].

Focusing on the electrochemical properties, closo-borane frameworks are known as chemically stable electron-accepting clusters, and their redox potentials can be tuned by substituent design [27]. This behavior is also rooted in the unique electronic structure of boron clusters, where skeletal electrons are delocalized through multicenter bonding, allowing redox processes to occur while maintaining the robustness of the cluster framework. Based on this property, applications as proton-coupled electron transfer reagents and reduction mediators have been reported (Figure 2b [28,29]). Furthermore, derivatives incorporating crown ether moieties exhibit reversible changes in cavity size associated with redox processes, enabling selective capture and release of Li^+^ ions [30], and carborane layers formed on lithium metal surfaces function as stable interfacial layers, promoting Li^+^ conduction and suppressing dendrite formation [31].

As described above, closo-borane clusters possess high chemical stability based on the polyhedral frameworks, and their derivatives exhibit both optical and electrochemical properties, making them important materials in boron cluster chemistry.

### 2.2. All-Boron Fullerene (Borospherene)

Following the discovery of C_60_ fullerene, interest in boron fullerenes such as B_60_ increased; however, due to the electron-deficient nature of boron compared with carbon, formation of stable closed-shell structures analogous to carbon fullerenes has been considered difficult [32]. Subsequently, several all-boron fullerene structures such as B_80_ have been theoretically proposed [33,34,35,36,37], although their experimental isolation has not been achieved. Among these candidates, B_40_ represents a prototypical all-boron fullerene composed entirely of boron atoms in a hollow cage framework. B_40_, also known as borospherene, has been experimentally identified in the gas phase by mass spectrometry and photoelectron spectroscopy combined with theoretical calculations [9]. However, because bulk synthesis and isolation remain challenging, the structural and functional properties of all-boron fullerenes have been discussed based on theoretical predictions.

For B_40_, an all-boron fullerene, the electron-deficient nature, multicenter bonding, and delocalized cage orbitals of the boron framework are key factors governing its electronic and optical properties, as in closo-boranes and carboranes. Theoretical calculations indicate that B_40_ behaves as a molecular cluster with a finite HOMO–LUMO gap.

From the viewpoint of optical properties, charge transfer induced by metal modification or metal encapsulation can significantly alter the electronic structure of borospherene. These effects have led to proposed applications in nonlinear optical (NLO) responses, optical switching, and electro-optic materials (Table 1) [38,39]. Furthermore, the axial chirality reported for specific borospherene structures suggests that zero-dimensional boron clusters can exhibit symmetry- and chirality-dependent optical responses [40]. Thus, the optical properties of borospherene should be understood in terms of finite-size molecular responses as well as the characteristic electronic structure, cage symmetry, structural distortion, and chirality of boron clusters.

**Figure 3:**
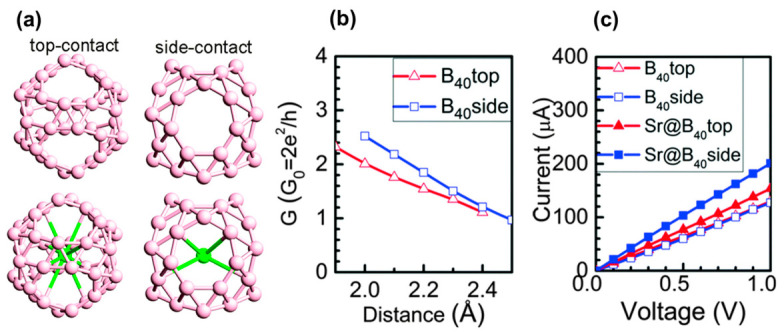
(**a**) Top and side views of isolated B_40_ (upper row) and Sr@B_40_ (lower row); (**b**) evolution data of conductance for the two models of B_40_ fullerene nanojunctions; (**c**) the calculated *I*–*V* curves of B_40_ and Sr@B_40_ nanojunctions in the two contact structures. Adapted with permission from Ref. [42]. Copyright 2016, Royal Society of Chemistry.

From the viewpoint of electrical and electrochemical properties, B_40_ has been theoretically predicted to show flexible redox behavior, reflecting both electron-accepting and electron-donating characteristics depending on the interacting species. This electronic flexibility enables stable adsorption of Na species, suggesting its potential as an anode material for sodium-ion batteries [41]. In contrast, closo-borane and carborane derivatives have been discussed in terms of Li^+^ capture and transport, as described above [30,31]. Therefore, these ion-related properties should be distinguished according to the relevant ion species, boron framework, and functional mechanism. Theoretical transport studies have also shown that B_40_ and Sr@B_40_ molecular junctions exhibit linear *I*–*V* characteristics and pronounced conductance, suggesting their potential as building blocks for molecular electronic devices (Figure 3a–c) [42].

At present, stable isolation methods and large-scale synthetic routes have not yet been established, limiting experimental applications. Nevertheless, improved synthetic accessibility and structural control could enable borospherene-based functional nanomaterials for batteries and molecular electronics.

## 3. One-Dimensional Materials

In contrast to zero-dimensional materials represented by boron clusters, one-dimensional materials form continuous frameworks along one direction and exhibit anisotropic properties. In this section, boron nanowires and boron nanotubes are discussed.

### 3.1. Boron Nanowire

Boron nanowires have been synthesized by various methods such as chemical vapor deposition (CVD) [10], magnetron sputtering [43], and laser ablation [44]. Depending on the synthesis conditions, their morphology, crystal structure, and crystallinity vary significantly. On silicon substrates, inclined and fused structures have been reported due to interactions between low-melting boron oxide and Au liquid metal catalysts [45]. In contrast, B–B-bonded cluster chains and one-dimensional boron nitride nanowires have been proposed theoretically [46,47]. These theoretical studies provide possible structural models for one-dimensional boron-based systems, but further experimental validation is needed to assess their relevance to real materials and their properties.

**Figure 4:**
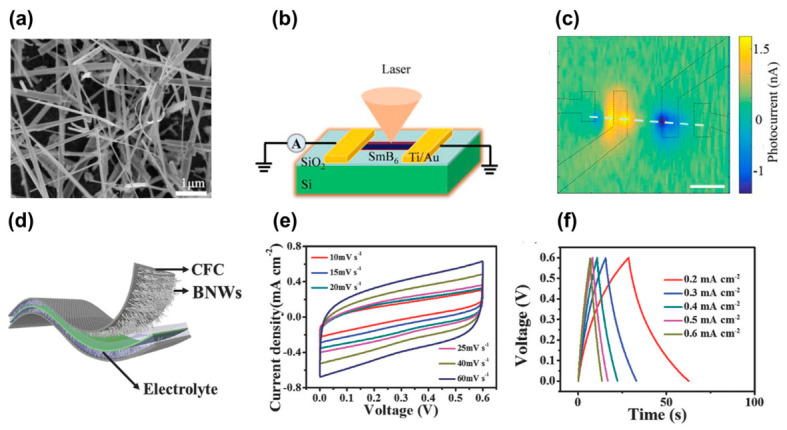
(**a**) Representative SEM images of SmB_6_ nanowires; (**b**) schematic image of the scanning photocurrent measurement setup for a SmB_6_ nanowire; (**c**) scanning photocurrent mapping of the SmB_6_ nanowire device under excitation (80 μW, 633 nm), measured at room temperature. The black and white dashed lines in (**c**) denote the outlines of the electrode and nanowire, respectively. The scale bar: 2 μm. Adapted with permission from Ref. [49]. Copyright 2018, AIP Publishing. (**d**) schematic illustration of a flexible all-solid-state supercapacitor based on the boron element nanowires–carbon fiber cloth electrodes. (CFC; carbon fiber cloth, BNWs: boron element nanowires); (**e**) cyclic voltammetry profiles of boron element nanowires–carbon fiber cloth devices at different scan rates (10–60 mV·s^−1^); (**f**) galvanostatic charge−discharge data of BNWs-CFC devices at different current densities. Adapted with permission from Ref. [55]. Copyright 2018, John Wiley and Sons.

Experimentally verified optical functions include anisotropic light scattering and second harmonic generation (SHG) under femtosecond laser irradiation [48]. These responses can be related to the one-dimensional morphology of the nanowires, the high refractive index and semiconducting electronic structure of boron, and possible direction-dependent polarizability or local symmetry breaking in the crystalline nanowire framework. SmB_6_ nanowires have also been demonstrated as broadband self-powered photodetectors, where the photocurrent originates from built-in electric fields at SmB_6_/Au interfaces rather than from elemental boron frameworks alone (Figure 4a–c) [49].

From the viewpoint of electrical and electrochemical properties, boron nanowires exhibit excellent field-emission (FE) characteristics, including low turn-on electric fields and high current durability, mainly because of their large aspect ratio and local electric-field enhancement at the nanowire tips [50,51,52,53]. High capacitance and durability under acidic, neutral, and alkaline conditions have also been reported for boron nanowire–carbon fiber cloth electrodes, suggesting potential applications as supercapacitor materials (Figure 4d–f) [54,55]. This performance probably arises from the combined effects of nanowire morphology, accessible surface area, the conductive carbon fiber substrate, and electrolyte-dependent charge-storage kinetics.

Thus, distinguishing intrinsic boron-framework effects from morphology-, surface-, and interface-derived contributions remains important for understanding one-dimensional boron-based materials.

### 3.2. Boron Nanotube

Boron nanotubes (BNTs) have been theoretically predicted to be stable [56,57]. In 2004, the growth of single-walled BNTs was experimentally confirmed on Mg-MCM-41 catalysts [58].

The optical properties of BNTs have been discussed based on theoretical calculations. Owing to their one-dimensional tubular structure and electron-deficient boron framework, BNTs are predicted to exhibit strong optical anisotropy, with dielectric constants, absorption coefficients, loss functions, and optical conductivity depending strongly on the polarization direction. Hexagonal BNTs exhibit metallic optical responses, with a maximum static dielectric constant of 60.38 and an absorption coefficient of 3.63 × 10^5^ cm^−1^ [59]. In contrast, ultrathin single-walled BNTs such as the (4,2) type exhibit semiconducting behavior with a direct bandgap of 0.40 eV [60].

**Figure 5:**
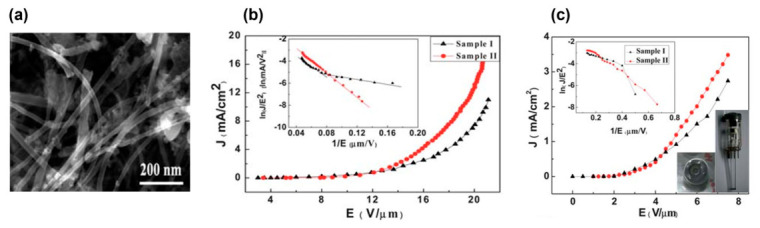
(**a**) An SEM image of one-dimensional boron nanostructures; (**b**) the current density vs. electric field curves. Samples I and II are samples prepared using different heating protocols. The inset is the FN plots; (**c**) the characteristic curves of field emission current versus the electric field applied to the extracting gate in a luminescent tube. Left-hand inset corresponds to their FN plots. Right-hand insets show digital camera photos of the nanostructured cathode in a stainless-steel substrate and the luminescent tube. Adapted with permission from Ref. [64]. Copyright 2010, Royal Society of Chemistry.

The electrical properties of BNTs have also been examined theoretically. Several studies suggest that BNTs possess metallic density of states (DOS) [61,62], and some models predict chirality-independent metallic behavior, unlike carbon nanotubes [63]. This feature is attributed to the electron deficiency and multicenter bonding of boron, which promote delocalized electronic states in the tubular framework. Experimentally, high conductivity and field-emission characteristics have been reported for BNT-related materials (Figure 5a–c) [64], supporting their potential as one-dimensional nanoelectronic materials. However, because experimental samples may contain defects, diameter distributions, oxidation, catalyst residues, and bundled structures, direct comparison with ideal theoretical models remains difficult. Further controlled synthesis and systematic property measurements are therefore required to establish reliable structure–property relationships for BNTs.

## 4. Two-Dimensional Materials

Borophene, a monolayer material composed entirely of boron, has been synthesized on Ag (111) substrates [11]. Multiple structures such as the β_12_ phase and χ^3^ phase have been identified. Theoretically, many phases exhibit metallic electronic structures [65,66,67]. In addition to these metallic phases, Pmmn borophene, often discussed as 8-Pmmn borophene, has been described as a semimetallic phase with highly anisotropic Dirac-type electronic dispersion [68]. This diversity indicates that the electronic structure of borophene is strongly governed by atomic arrangement, buckling, and vacancy patterns.

**Figure 6:**
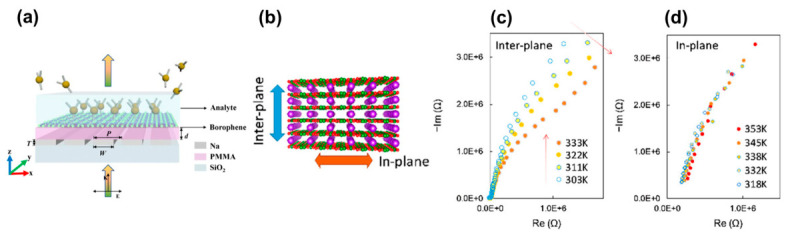
(**a**) Schematic diagram of the proposed borophene-based hybrid plasmonic structure. Reproduced from Ref. [70] under the terms of the Creative Commons Attribution 4.0 International License. (**b**) measurement directions of the borophene oxide crystal structure; (**c**,**d**) Nyquist plots of the impedance measurements at several temperatures along (**c**) interplane (The red arrow indicates the direction toward higher temperatures.) and (**d**) in-plane directions. Adapted with permission from Ref. [77]. Copyright 2019, American Chemical Society.

From the viewpoint of optical properties, borophene has been theoretically predicted to exhibit characteristic optical responses derived from its electron-deficient boron framework, multicenter bonding, and structural anisotropy. In particular, in-plane anisotropic absorption and plasmonic responses have been investigated by theoretical calculations (Figure 6a [69,70]). These properties are closely related to the anisotropic distribution of delocalized carriers in boron networks, highlighting the importance of boron-specific electronic structures rather than generic two-dimensional effects. In addition, borophene-based mixed-dimensional heterostructures have recently been explored as photoelectric platforms; for example, 0D Bi nanoparticle/2D borophene van der Waals heterojunctions can promote photoinduced carrier separation through interfacial charge transfer and built-in electric fields [71].

From the viewpoint of electrical properties, borophene has also been theoretically predicted to show high carrier mobility and tunable electronic structures [72]. Hydrogen termination, strain, and external electric fields have been proposed as effective methods for modulating its band structure, including metal–semiconductor transitions [73,74,75,76]. Experimentally, however, verified borophene-based materials remain limited because pristine borophene is sensitive to substrates, termination, and external environments. As an experimentally accessible derivative, oxidized borophene synthesized in solution forms ionic layered crystals composed of boron–oxygen atomic layers and interlayer cations, and exhibits anisotropic electrical behavior, with semiconducting transport in the interplane direction and metal-like transport in the in-plane direction of the boron network (Figure 6b–d [77]). Liquid-crystalline derivatives of oxidized borophene further demonstrate that chemically stabilized borophene-like layered frameworks can be developed into functional optical and dielectric materials [78,79]. Metalloborophenes also expand the structural diversity of boron-based two-dimensional materials, because metal–boron bonding provides an additional parameter for tuning electronic states and related functions [80].

Beyond electronic and optical functions, borophene-based materials have recently attracted attention for hydrogen-related energy applications, including the hydrogen evolution reaction [81]. These studies indicate the broad potential of boron-based two-dimensional materials for optoelectronic, energy-conversion, and electrochemical applications, although systematic comparison between theoretical predictions and experimental observations remains essential for clarifying their intrinsic functions.

## 5. Discussion

As summarized in Table 2, dimensionality provides a useful basis for comparing the electronic structures and optical/electrical responses of boron-based low-dimensional nanomaterials.

A cross-dimensional comparison shows that structural differences among 0D closed cage-like frameworks, 1D axially continuous frameworks, and 2D extended in-plane networks are reflected in electron confinement, directional transport pathways, and in-plane electronic delocalization, respectively. In boron frameworks characterized by multicenter bonding, electronic distribution and orbital overlap strongly depend on the framework structure; therefore, dimensionality serves as an important structural factor [7,8,9,10,11]. At the same time, even within the same dimensional class, electronic structures and transport pathways can be further modulated by termination, size, and external environments.

In zero-dimensional boron clusters, confinement of electrons within finite cage frameworks gives rise to discrete frontier orbitals. Therefore, the optical and redox properties of borane cluster and all-boron fullerene systems can be understood as molecular-level responses controlled by cage topology and chemical modification [12,13,14,15,16,17,18,19,32,33,34,35,36,37]. For example, ICT emission and photochromism in carborane derivatives originate from charge transfer between donor units and electron-accepting boron-rich cages [20,21,22,23,24,25,26]. In addition, redox tuning enables electron-transfer mediation, Li^+^ trapping, and interfacial stabilization in lithium metal batteries [27,28,29,30,31]. In borospherenes, metal modification or endohedral encapsulation perturbs the delocalized cage orbitals, leading to predicted nonlinear optical responses and molecular conductance [38,39,40,41,42].

In one-dimensional boron nanowires and boron nanotubes, the boron framework extends continuously along one direction. This axial continuity forms directional pathways for charge transport and light–material interactions [10,43,44,45,46,47]. Thus, anisotropic light scattering, second-harmonic generation, and field emission in boron nanowires can be explained by their elongated morphology, direction-dependent polarizability, and local electric-field enhancement at the nanowire tips [48,50,51,52,53]. Their electrochemical performance also reflects accessible surface area, contact with conductive substrates, and interfacial effects [54,55]. In boron nanotubes, diameter, curvature, and chirality further modulate orbital overlap, electronic structure, and optical anisotropy [56,57,58,59,60,61,62,63,64].

In two-dimensional boron systems, extended in-plane boron networks allow electronic delocalization over the entire sheet. The metallic behavior of β_12_ and χ^3^ borophene originates from vacancy arrangements that stabilize electron-deficient sheets, whereas the anisotropic buckled structure of 8-Pmmn borophene gives rise to direction-dependent Dirac-type dispersion [65,66,67,68]. These band-like electronic structures account for plasmonic responses, polarization-dependent optical absorption, and high carrier mobility [69,70,71,72]. Hydrogen termination, strain, and external electric fields can further tune the band structure by modifying electron count and B–B orbital overlap [73,74,75,76]. In oxidized borophene crystals, anisotropic electrical transport can be understood on the basis of in-plane boron–oxygen networks and interlayer cations [77].

These cross-dimensional comparisons indicate that dimensionality is a key structural factor for understanding boron-based nanomaterials. However, dimensionality alone does not determine their properties; size effects, bonding topology, edge structures, defect density, and local chemical environments can also modify orbital distribution, band dispersion, and transport pathways. Therefore, future studies should clarify intrinsic structure–dimension–size–property relationships through systematic studies of structurally controlled materials, supported by appropriate theoretical and experimental analyses. For 0D systems, experimentally validated correlations between cluster structure and optical or redox responses are required. For 1D systems, intrinsic boron-framework effects should be distinguished from morphology-, surface-, and interface-derived contributions. For 2D systems, chemically stable and structurally defined materials are needed to evaluate intrinsic optical, electrical, and optoelectronic functions. Establishing these relationships will provide rational design principles for boron-based nanomaterials with tunable optical and electrical properties.

## 6. Conclusions

In this review, boron-based low-dimensional materials were systematically discussed according to dimensionality, with their optical and electrical properties related to structural factors such as termination, size, and external environments. This organization clarifies how their electronic structures and functions evolve from zero-dimensional clusters to one-dimensional nanostructures and two-dimensional sheets. Zero-dimensional systems exhibit charge-transfer emission, redox activity, and nonlinear optical responses, whereas one-dimensional systems show directional charge transport, anisotropic optical responses, and field-emission behavior arising from axial structural continuity. In two-dimensional systems, extended boron networks give rise to plasmonic responses, carrier transport, and optical anisotropy, which can be further modulated by vacancy arrangements, buckling, termination, strain, oxidation, and interlayer environments. These comparisons indicate that dimensionality is a key structural factor for understanding boron-based low-dimensional materials; however, their properties are also strongly affected by termination, size, and external environments. Despite these structural variations, the electron-deficient character and multicenter bonding of boron provide a common chemical basis for these diverse dimension-dependent functions. Establishing clear relationships among dimensionality, structural features, and optical/electrical properties will provide rational design of boron-based low-dimensional materials with tunable optoelectronic and energy-related properties. These design principles will facilitate the development of boron-based materials for practical optoelectronic and energy applications.

## Figures and Tables

**Table 1:** Nonlinear optical properties of Li_3_@B_40_ and Li_3_@C_60_ systems. Average linear polarizability ($\bar{\alpha }$), first hyperpolarizability (β), and second hyperpolarizability ($\gamma_{∥}$) of Li_3_@B_40_ and Li_3_@C_60_ systems. Based on Ref. [39].

NLO Property	Li_3_@B_40_	Li_3_@C_60_
$\bar{\alpha }$	554.2	584.7
β	129.4	79.9
$\gamma_{∥}$	3.6 × 10^5^	2.1 × 10^5^

**Table 2:** Structure–property relationships of boron-based low-dimensional nanomaterials as a function of dimensionality.

Dimension	0	1	2
**Examples**	Borane cluster, All-boron Fullerene	Nanowire, Nanotube	Borophene or borophene derivatives
**Electronic structure**	Molecular	Metallic or semiconducting	Metallic or semiconducting *
**Example of** **electronic feature**	Redox activity	Efficient electron emission	Possible superconductivity (theoretical prediction), Semiconductivity
**Example of** **optical feature**	ICT emission, NLO response	Anisotropic	Plasmonic, Anisotropic
**Dominant** **structural factor**	Number of atoms, Cage topology, etc.	Diameter, Width, Curvature, etc.	Vacancy pattern, Counter cations, etc.

* Most borophene phases are metallic, while semiconducting behavior can emerge in selected modified or functionalized systems (borophene derivatives).

## Data Availability

No new data were created or analyzed in this study. Data sharing is not applicable to this article.
